# Diphenyl Urea Derivatives as Inhibitors of Transketolase: A Structure-Based Virtual Screening

**DOI:** 10.1371/journal.pone.0032276

**Published:** 2012-03-05

**Authors:** Cristian Obiol-Pardo, Gema Alcarraz-Vizán, Marta Cascante, Jaime Rubio-Martinez

**Affiliations:** 1 Departamento de Química Física, Facultat de Química, Universitat de Barcelona and Institut de Recerca en Química Teòrica i Computacional (IQTCUB), Barcelona, Spain; 2 Departamento de Bioquímica i Biologia Molecular, Facultat de Biologia, Universitat de Barcelona and Institute of Biomedicine at Universitat de Barcelona (IBUB), Barcelona, Spain; Spanish National Cancer Center, Spain

## Abstract

Transketolase is an enzyme involved in a critical step of the non-oxidative branch of the pentose phosphate pathway whose inhibition could lead to new anticancer drugs. Here, we report new human transketolase inhibitors, based on the phenyl urea scaffold, found by applying structure-based virtual screening. These inhibitors are designed to cover a hot spot in the dimerization interface of the homodimer of the enzyme, providing for the first time compounds with a suggested novel binding mode not based on mimicking the thiamine pyrophosphate cofactor.

## Introduction

Transketolase (EC 2.2.1.1) is a homodimeric enzyme that catalyses the reversible transfer of two carbons from a ketose donor substrate to an aldose acceptor substrate. Transketolase is the most active enzyme involved into the non-oxidative branch of the pentose phosphate pathway [1], in charge of generating the ribose molecules necessary for nucleic acid synthesis. Together with the finding that this pathway is highly expressed in the cancer cell [2], this enzyme provides an excellent target for novel chemotherapeutic agents. Additionally, several crystal structures of this enzyme are available [3], [4], [5], [6], [7] and notably, the human variant of transketolase was recently reported as well [8] allowing the rational structure-based design of human inhibitors.

The active centre of transketolase contains a thiamine pyrophosphate cofactor, coordinated to a divalent metal ion, whose binding site has been used for the development of enzyme inhibitors. The most representative inhibitors that mimetize the interactions of thiamine pyrophosphate are oxythiamine [9] and thiamine thiazolone diphosphate [10]. Unfortunately, these compounds lack selectivity as thiamine pyrophosphate is a common cofactor found in multiple enzymes, such as pyruvate dehydrogenase [11]. More recently, several thiamine antagonists were designed with the aim of obtaining more selective inhibitors with improved physical properties [12], [13], [14]. Nonetheless, it is interesting to find additional binding sites allowing drug discovery, not based on the active centre of transketolase but on critical allosteric points of the enzyme. Here, we utilize the homology model of human transketolase recently reported by our group [15] to analyze the hot spot residues of the homodimeric interface and perform a pharmacophore-based virtual screening. This strategy yielded a novel family of compounds, containing the phenyl urea group, as new transketolase inhibitors not based on antagonizing thiamine pyrophosphate. The activity of these compounds, confirmed in transketolase cell extract and in two cancer cell lines, suggests that the phenyl urea scaffold could be used as novel starting point to generate new promising chemotherapeutic agents by targeting human transketolase.

## Results and Discussion

### Virtual screening protocol

The homology model of human transketolase [15] (see Materials and Methods section) was used to analyze the most stable contacts belonging to the dimer interface of the enzyme. It is known that the active centre of transketolase containing thiamine pyrophosphate is stabilized by contacts of the two subunits [8] and thereby transketolase activity is closely related with its dimer stability.

**Figure 1:**
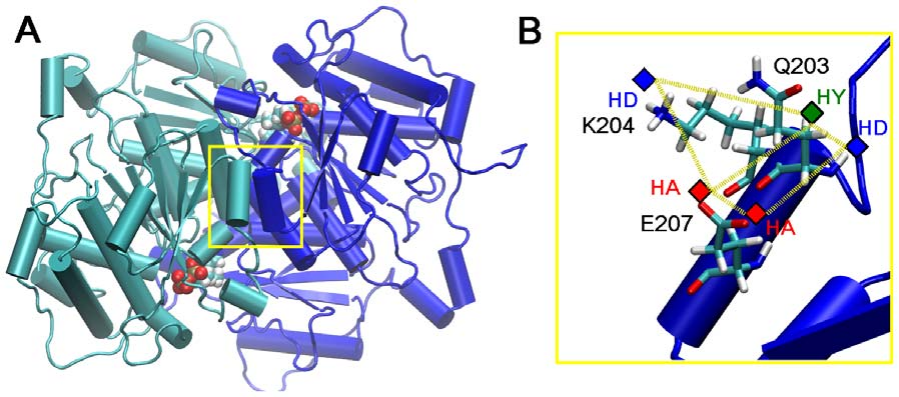
Transketolase structure model. A) Homology model of human transketolase showing the antiparallel alpha helices involved in dimerization. B) Close view of the alpha helix D200-G210 showing the selected residues of the 5-point pharmacophore. HY: hydrophobic contact, HA: hydrogen acceptor, HD: hydrogen donor.

The dimer interface was evaluated through molecular dynamics simulations [15] (see Materials and Methods section) calculating the interaction energies between all residues of both monomers to conclude that the conserved sequence D200-G210 fulfils the criteria used for pharmacophore selection. The high sequence conservation of D200-G210 with respect to the template (50%) [15] was considered an important trend that could point to an area of dimer stabilization. This short sequence belongs to an alpha helix motif that interacts with the same fragment of the partner monomer forming the antiparallel alpha helices structure shown in Figure 1A. This sequence forms a hydrogen bond donor between the amino group of Q203, of the first monomer, and the oxygen atom of the carboxylate of E207, belonging to the second monomer. Carboxylate of E207 of the first monomer forms two hydrogen bond acceptors, with Q203 and K204 of the second subunit. Finally, terminal amino of K204 of the first monomer maintains a hydrogen bond donor with the carboxylate of E207, of the second monomer. On the other hand, the analysis of van der Waals energies revealed us that Q203 offers a major contribution when interacting with the fragment D200-G210, providing around −8 kcal/mol and that residues K204 and E207 provided high electrostatic energies (around −20 kcal/mol).

Accordingly, this alpha helix sequence was utilized to configure a 5-point pharmacophore (Figure 1B) to perform a structure-based virtual screening (see Materials and Methods section). This process yielded 128 candidate molecules with a structure able to accommodate the five interactions shown in the natural protein sequence, and therefore with the potential ability to function as dimerization inhibitors (Supplementary Material, Table S1). After that, a docking procedure (see Materials and Methods section) was carried out to refine the hit selection from the pool of candidates applying a geometrical criterion (RMSd-based) and consensus scoring employing the XSCORE function [16]. Best ranked compounds were visually inspected and seven of them (named T1–T7) were purchased for experimental validation.

### *In vitro* enzymatic inhibition of transketolase and cell viability inhibition

**Figure 2:**
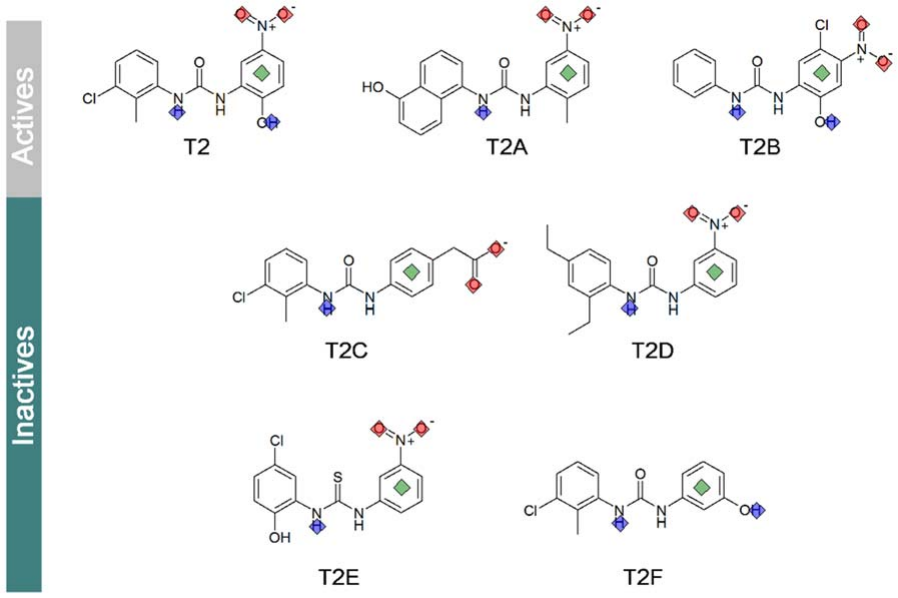
Structures of the reported diphenyl urea derivatives. In the upper part, compounds which showed good inhibitory activity. In the bottom part, compounds which showed poor activity. The pharmacophoric points are also shown; in red for hydrogen acceptor points, in blue for hydrogen donor points and in green for hydrophobic points.

Most promising compounds were purchased as potential transketolase inhibitors and tested for enzyme activity using spectrophotometric and fluorimetric methods (see activity of two of them in Supplementary Material Figure S1). Among the products tested for transketolase activity, the compound showing the best inhibitory action (T2, Figure 2) was selected to perform additional cell proliferation assays (T1, T3, T5, T6 and T7 were inactive in the enzymatic assay and T4 was too hydrophilic to penetrate into the cells, so all of them were discarded). This compound presented an IC_50_ of 10 µM in HT29 and 9 µM in HCT116 (see Materials and Methods section) (Table 1). For comparison purposes, we also assessed the effect of oxythiamine, a reference transketolase cofactor antagonist, resulting in a much higher IC_50_ of 8 mM in both cell types.

**Table 1:** Activities of the reported diphenyl urea derivatives.

IC_50_ (µM)	Transketolase from cell extract	Cell viability (HT29/HCT116 cells)
T2	200	10/9
T2A	150	15/17
T2B	100	6/12
T2C	>>200	200/200
T2D	>>200	>200/>200
T2E	>>200	20/13
T2F	>>200	na

### Compound T2 derivatives and structure-activity analysis

Following the encouraging results of compound T2, a second generation of diverse derivative molecules was purchased in order to improve the inhibition activity. The docked structure of compound T2 was considered here as a starting point to visualize structural changes able to improve the interaction pattern.

The SciFinder (ACS) tool was used to search for commercially available T2 derivatives and resulted in five most promising compounds (T2A, T2B, T2C, T2D and T2E), whose binding mode to transketolase was evaluated throughout docking and scoring (see Materials and Methods section and Supplementary Material Table S2). Additionally a compound not fulfilling the pharmacophore was also purchased as a negative control (compound T2F).

For the six compounds (Figure 2), an equivalent transketolase inhibition test was performed, varying their concentration in the range of 50 to 300 µM. The effect of these compounds over cell proliferation was also studied in HT29 and HCT116 cell lines. Results are summarized in Table 1. The thiourea derivative T2E was poorly active confirming that the carbonyl group is essential for transketolase inhibition. The negative control (compound T2F) which lacks the fundamental nitro group showed no activity, as expected seeming to confirm the suggested binding site. Compound T2C which presents a carboxyl group as mimetic of the nitro moiety was also inactive. Compound T2D showed no inhibition of transketolase, suggesting that the *meta* ethyl groups in the aromatic ring are probably too bulky or too hydrophobic for maintaining a suitable binding. Conversely, compounds T2A and specially T2B (Supplementary Material Figure S2), which are more similar to the parent compound T2, showed a lower IC_50_ both in the transketolase inhibitory activity experiment and in the cell viability assay (Table 1).

Both compounds can be considered as promising hits for developing further human transketolase inhibitors.

### Proposed binding mode and interaction energies of compound T2 and its derivatives

**Figure 3:**
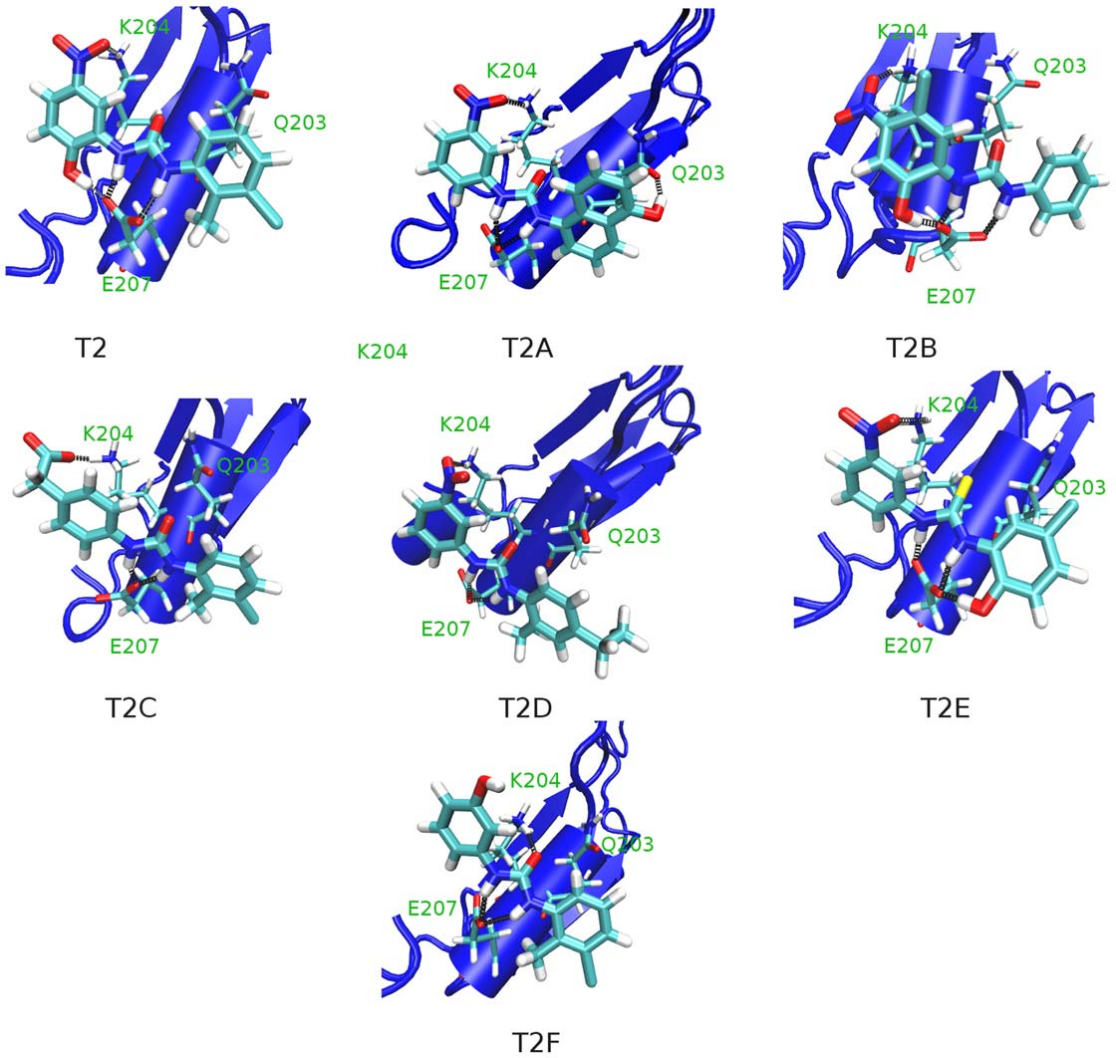
Proposed binding mode of compound T2 and its derivatives (T2A–T2F). Black dots represent hydrogen bonds.

Figure 3 shows the proposed binding mode of the compound T2 and its derivatives, and Table 2 lists the ligand-protein interaction of the compounds extracted from the force field and also the binding energies by applying an standard MMPBSA protocol (see Materials and Methods) upon the minimized structures. In compound T2 the nitro group interacts strongly with K204 by forming a hydrogen bond, the two urea amino groups form two hydrogen bonds with E207. Additionally, the hydroxyl group of T2 also forms a third hydrogen bond with E207. Finally, the two phenyl rings establish van der Waals contacts, mainly with the hydrophobic part of side chains of Q203 and K204 (Figure 3). All in all, the interactions of this compound with the alpha helix sequence resemble remarkably the protein-protein recognition of the two alpha helices in the homodimer of transketolase. Moreover the proposed binding mode could explain the inactivity of compounds T2F and T2C, since they lack the obligatory nitro group. Table 2 shows that the electrostatics energies of the nitro group are in between −22 and −25 kcal/mol; T2F binding lacks this contribution and the carboxylate of T2C only offers −10 kcal/mol.

**Table 2:** Ligand-receptor interactions, contribution of the nitro group and MMPBSA energy for all compounds.

Compound	Ligand-receptor interactions	Electrostatic intermolecular energy of the nitro group (kcal/mol)^1^	MMPBSA energy (kcal/mol)^2^
T2	Urea amino groups-E207 (HB)Hydroxyl-E207 (HB)Nitro-K204 (HB)m-chlorophenyl-Q203 (VDW)	−20	−25.03
T2A	Urea amino groups-E207 (HB)Nitro-K204 (HB)Hydroxynaphtalene-Q203 (HB)Hydroxynaphtalene-Q203 (VDW)	−22	−15.21
T2B	Urea amino groups-E207 (HB)Hydroxyl-E207 (HB)Nitro-K204 (HB)Phenyl-Q203 (VDW)	−25	−15.66
T2C	Urea amino groups-E207 (HB)Carboxylate-K204 (HB)o-methyl,m-chlorophenyl-Q203 (VDW)	−10 (carboxylate)	−14.67
T2D	Urea amino groups-E207 (HB)Nitro-K204 (HB)o,p-diethylphenyl-Q203 (VDW)	−25	−12.16
T2E	Urea amino groups-E207 (HB)Hydroxyl-E207 (HB)Nitro-K204 (HB)o-hydroxy,m-chlorophenyl-Q203 (VDW)	−25	−19.05
T2F	Urea amino groups-E207 (HB)carbonyl-K204 (HB)o-methyl, m-chlorophenyl-Q203 (VDW)	none	−14.53

HB denotes for hydrogen bond formation, VDW denotes for van der Waals interaction.

1 : Extracted from the electrostatic term of the force field.

2 : MMPBSA energy without computing the entropy contribution to binding.

The bulky sulphur of the thiourea moiety of compound T2E also affected negatively the binding affinity although the MMPBSA interaction energies remained similar to the parent compound T2 (Table 2). It is possible that the inactivity of T2E is more related with a lower solubility of the compound. It is worth mentioning that, except T2E, the MMPBSA energies are in accordance with the experimental activities, being higher in the active compounds (T2, T2A, T2B) as compared with the inactive ones T2C, T2D and T2F.

### Comparison with the crystal structure of human transketolase

**Figure 4:**
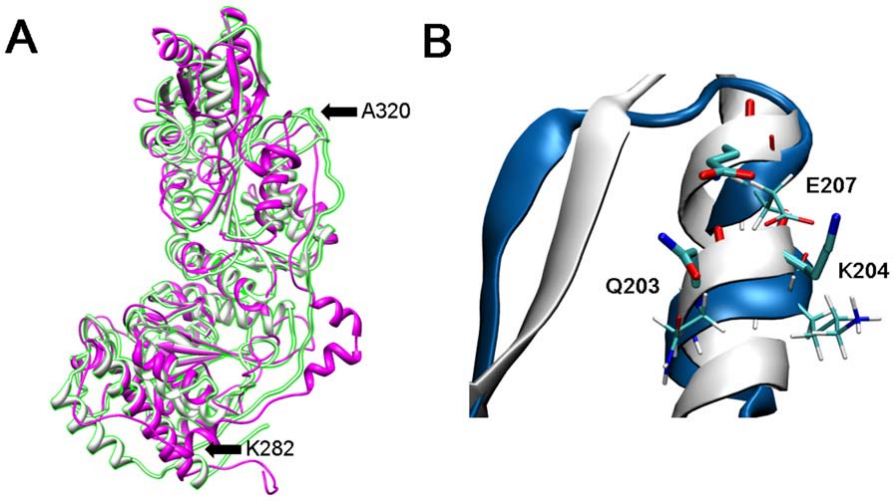
Comparison of transketolase model with crystal structure. A) Superimposition of the homology model of human transketolase (in pink) with the recently released crystal structure (in white). B) Close view of the residues used for pharmacophore definition, in the homology model (in pink and thin residues) and in the crystal structure (in white and thick residues).

During the course of this research, the crystal structure of human transketolase was made public (pdb code 3MOS) [8] allowing its comparison with our previously reported homology model [15] that was used in the virtual screening protocol. Figure 4A shows a superimposition of our initial homology model and the crystal structure of transketolase highlighting a high similarity with a backbone RMSd of only 1.1 Å (for modeled monomer 1 and 331 atom pairs) and 1.2 Å (for modeled monomer 2 and 330 atom pairs). Overall our model predicted correctly the main structural parts of the protein, although the uncommon long loop of transketolase found in K282-A320 was not well predicted (Figure 4A between arrows). Nonetheless, this sequence is solvent exposed not participating in dimer stabilization nor catalytical activity. It is worth mentioning that the proposed pharmacophore used in this study (formed by Q203, K204 and E207) can be also extracted, with minor distances differences (all atom RMSd of 1.77 Å and 1.82 Å for modeled monomer 1 and 2, respectively), from the crystal structure of human transketolase (Figure 4B). Thereby, either our model or the crystal structure should be considered equally representative for the alpha helix fragment used in our virtual screening protocol.

### Conclusions

Novel phenyl urea derivatives have been described as inhibitors of human transketolase, an excellent target for the advent of new anticancer drugs. Notably, the proposed binding mode of compounds involves interactions with the alpha helix sequence D200-G210 interfering likely with the enzyme dimerization.

Although the activity of the reported inhibitors is only in the micromolar range, their proposed novel binding site and binding mode can be used for the rational design of future more potent inhibitors of transketolase not based on mimicking the non drug-like thiamine pyrophosphate cofactor.

## Materials and Methods

### Virtual screening protocol

The homology model of human transketolase previously reported [15] was used to scan for the hot spots responsible for dimer stabilization. The model was energy-minimized and subjected to a molecular dynamics simulation (MD) using the Amber program [17] and the ff94 and GAFF force fields [18]. MD temperature was set to 300 K in increments of 30 K/10 ps and optimal density was reached by a NPT step of 40 ps. Then, the system was simulated in the NVT ensemble for 7 ns. Stabilization of total energy was obtained in the last 1.6 ns, that were considered, therefore, the production time and were used for the further analysis of interactions. Hydrogen bonds, van der Waals contacts and electrostatic interactions formed between the two transketolase monomers were monitored throughout the production time of the molecular dynamics simulation [15] extracting the van der Waals and electrostatics components of the force field. For this purpose, the energies of each residue of one monomer of transketolase against the complete other monomer were obtained (no cut-off was used). Those residues with higher values were visually inspected in order to deduce a protein-protein pharmacophore. In this stage not only the interaction energies were considered but also the type of interactions and the sequence conservation with respect to the template.

Subsequently, the pharmacophore was introduced as a hypothesis for database screening employing the Catalyst program (Accelrys Inc. CATALYST™) and the following libraries of commercially available compounds: Mini Maybridge, SPECS, Nat Diverse, ACD, IBS, NCI, Aurora and Derwent. Search concluded with 128 drug-like hits that accomplished the pharmacophore query with a deviation of 1 Å or less. These compounds were acquired and refined trough a docking-scoring protocol. Docking was performed with our home-made program Dock_Dyn [19] by imposing the pharmacophore constraint to all conformations. This process selects only those conformations that fulfill the pharmacophore restriction speeding up the docking process. First ranking of ligands was obtained by evaluating their pharmacophore RMSd compared with the protein pharmacophore. In order to account slightly for the protein flexibility, the atom radii of both the receptor and the ligands were reduced by 40%, allowing therefore a minor steric clash in the binding site. After that, the empirical-based scoring function XSCORE [16] was utilized to score and rank all docking solutions. Best ranked compounds according to RMSd and XSCORE criteria were visually inspected both in terms of pharmacophore deviation and ligand-protein interactions, and purchased for experimental validation. Moreover the intermolecular force field contributions of the nitro groups (or the equivalent carboxylate group) of the compounds were analyzed to qualitatively measure the differences in binding affinity. Additionally an MMPBSA protocol [20] was performed by taking the minimized docked structures. Entropy calculation was neglected assuming that it will remain constant among the derivatives due to their similarity.

### Purchase of compounds

Seven compounds were acquired from commercial suppliers in their highest purity (>80%): compound T2 (Sigma Aldrich; id = S715719-1EA), compound T2A (Salor Sigma; id = S847984), compound T2B (LaboTest Stock; id = LT03054002), compound T2C (Scientific Exchange Products; id = M-640547), compound T2D (Scientific Exchange Products; id = M-442855), compound T2E (Scientific Exchange Products; id = M-620773) and compound T2F (Sigma Aldrich; id = S867616). Positive ESI Mass Spectra results for all the studied compounds are included in the Supplementary material (Tables S3, S4, S5, S6, S7, S8, S9, S10).

### Cell culture

HCT116 human colon carcinoma cells (gift from Dr Capellà, Institut Català d'Oncologia, Barcelona, Spain) were grown in DMEM∶HAM F12 (1∶1), supplemented with 10% heat-inactivated foetal calf serum (FCS) (PAA Laboratories, Pasching, Austria), l-glutamine (2 mM), pyruvic acid (1 mM), non-essential aminoacids (1%) and antibiotics: 100 U×mL^−1^ penicillin and 100 µg×mL^−1^ streptomycin. HT29 human colon adenocarcinoma cells (obtained from the American Type Culture Collection -ATCC-) were grown in Dulbecco's Modified Eagle's Medium, 25 mM d-glucose, 4 mM l-glutamine supplemented with 10% heat-inactivated FCS, and antibiotics: 100 U×mL^−1^ penicillin and 100 µg×mL^−1^ streptomycin (Invitrogen, Paisley, UK). All cell cultures were carried out at 37°C in a humidified atmosphere with 5% CO_2_. Subconfluent monolayers of cells were used in all experiments.

### Preparation of cell extracts for enzyme activity studies

Cell cultures were washed with PBS, detached from the flasks using 0.025% trypsin-EDTA and then resuspended in lysis buffer (1 mM dithiothreitol, 1 mM EDTA, 0.02% Triton X-100, 0.02% sodium deoxycholate, 0.2 mM phenylmethylsulfonyl fluoride, 1% sodium azide and 20 mM Tris-HCl, pH 7.5). Cells were homogenized using a laboratory sonicator (1/2 Liter Branson 200 Ultrasonic bath, 5 min, 40 kHz, 4°C) and immediately ultracentrifuged at 105000×g for 1 hour at 4°C. The supernatant was separated and approximately 300 µg of protein cell extract used for the determination of transketolase enzyme activity using a Cobas Mira Plus chemistry analyzer (HORIBA ABX, Montpellier, France) or a spectrofluorimeter Kontron SFM-25 (Bio-Tek Instruments, VT, United States).

### Determination of transketolase activity

Transketolase (EC 2.2.1.1) activity was determined using the enzyme linked method described by Smeets and colleagues [21]. Briefly, cell extract and inhibitors were added to a cuvette containing 5 mM MgCl_2_, 0.2 U×mL^−1^ glycerophsphate dehydrogenase-triosephosphate isomerase (from rabbit muscle), 0.2 mM NADH, 0.1 mM thiamine pyrophosphate (TPP) and 50 mM Tris-HCl, pH 7.6 at 37°C. The reaction was initiated by the addition of 300 µL substrate mixture (see below) per mL of cuvette volume. The oxidation of NADH, which is directly proportional to transketolase activity, was followed by the decrease in absorbance at 340 nm (spectrophotometric method) or in fluorescence at 450 nm when excited at 330 nm (fluorimetric method) before and after the addition of the different compounds tested. A blank with all the assay components but the sample was performed in each experiment by measuring the oxidation of NADH. The substrate mixture was prepared by dissolving 50 mM ribose-5-phosphate in 50 mM Tris-HCl, pH 7.6, and adding 0.05 IU ribulose-5-phosphate-3-epimerase (from baker's yeast) and 0.25 IU phosphoriboisomerase (from spinach). The substrate mixture was continuously stirred and heated for 1 hour at 37°C and then kept at −20°C until use.

Protein concentration of cell extracts was determined using the BCA Protein Assay (Pierce Biotechnology, Rockford, IL) to calculate the specific activity of the transketolase enzyme.

### MTT assay

Cell viability was determined using the MTT assay, which is based on the ability of live cells to cleave the tetrazolium ring, thus producing formazan, which absorbs at 570 nm. 5000 cells/well HT29 cells or 1700 cells/well HCT116 cells were seeded into 96-well plates and incubated for 24 h prior to the addition of the different compounds at different concentrations, in triplicate. After incubation for 72 h, the supernatant was aspirated and 100 µL of filtered MTT (0.5 mg/mL) was added to each well. Following 1 h of incubation, the supernatant was removed and the formazan precipitated was dissolved in DMSO. Relative cell viability was measured by absorbance at 550 nm on an ELISA plate reader (Tecan Sunrise MR20-301, TECAN, Austria). Concentrations that inhibited cell growth by 50% (IC_50_) after treatment were determined.

## Supporting Information

Figure S1 Transketolase activity in cell extracts after T2 and T4 incubation using spectrophotometric and fluorimetric methods.
(PDF)

Figure S2 Transketolase activity in cell extracts after T2A and T2B incubation (upper panels). Dose response curves of cell viability after 72 h T2B incubation in HT29 and HCT116 cells (lower panels).
(PDF)

Table S1 Smiles and MDL numbers of best ranked molecules.
(XLS)

Table S2 XSCORE and pharmacophore RMSd of T2 and T2 derivatives.
(XLS)

Table S3 Positive ESI Mass Spectra results for T2 compound.
(PDF)

Table S4 Positive ESI Mass Spectra results for T2A compound.
(PDF)

Table S5 Positive ESI Mass Spectra results for T2B compound.
(PDF)

Table S6 Positive ESI Mass Spectra results for T2C compound.
(PDF)

Table S7 Positive ESI Mass Spectra results for T2D compound.
(PDF)

Table S8 Positive ESI Mass Spectra results for T2E compound.
(PDF)

Table S9 Positive ESI Mass Spectra results for T2F compound.
(PDF)

Table S10 Positive ESI Mass Spectra analysis. Accurate mass of [M+H]^+^ ions.
(TIF)
