# Supplementary figures and images for: Diphenyl Urea Derivatives as Inhibitors of Transketolase: A Structure-Based Virtual Screening
Source: PLoS One. 2012 Mar 5;7(3):e32276. doi: 10.1371/journal.pone.0032276 (PMC3293897; doi:10.1371/journal.pone.0032276)

**Figure S2**

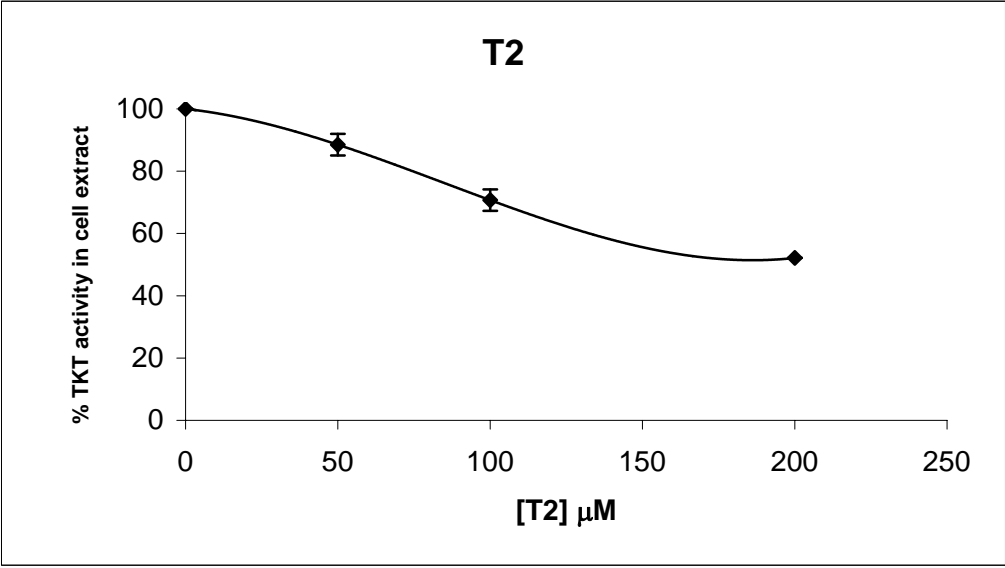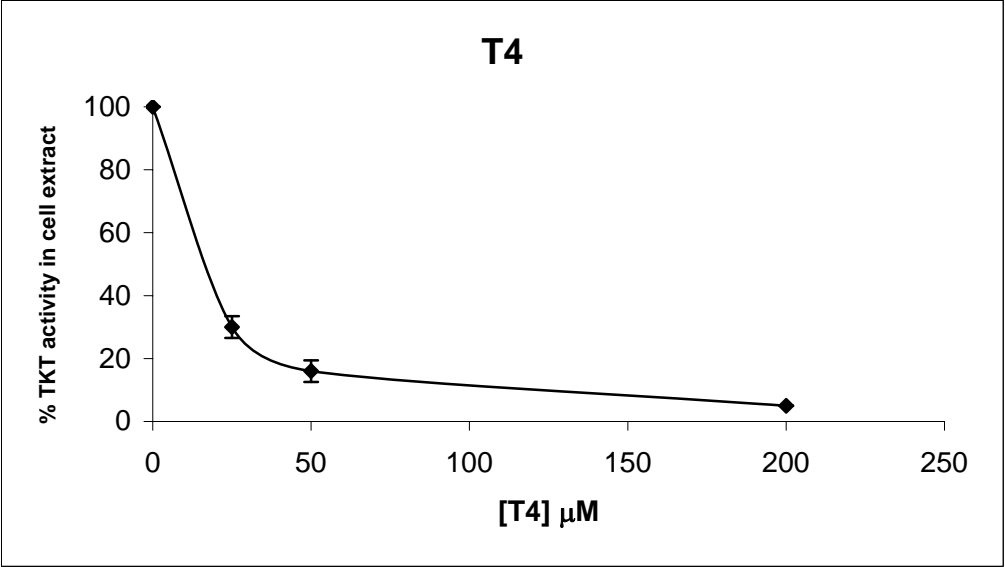

Supplement: Figure S1 — Transketolase activity in cell extracts after T2 and T4 incubation using spectrophotometric and fluorimetric methods. (PDF) [file pone.0032276.s001.pdf]

**Figure S4**

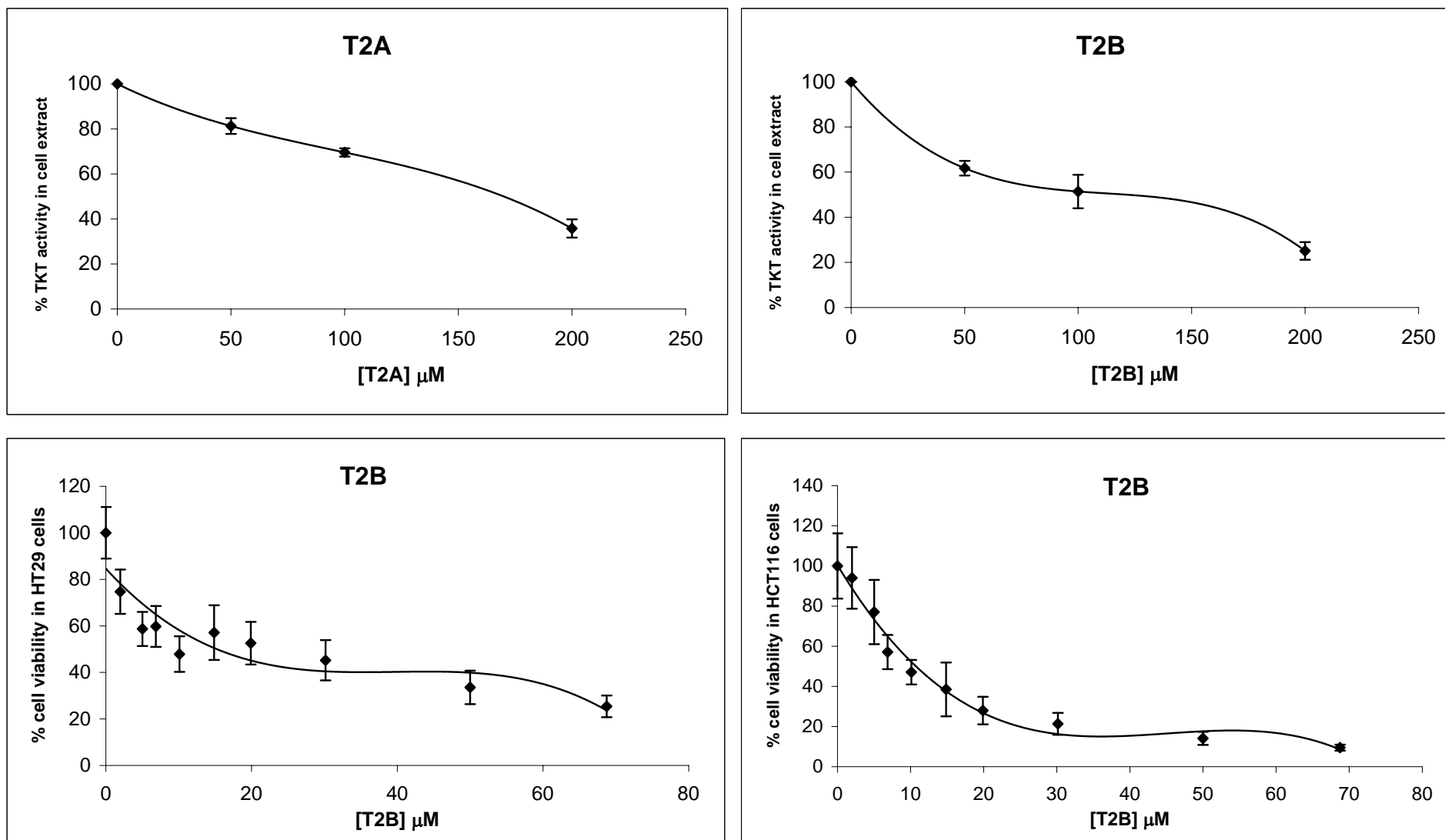

Supplement: Figure S2 — Transketolase activity in cell extracts after T2A and T2B incubation (upper panels). Dose response curves of cell viability after 72 h T2B incubation in HT29 and HCT116 cells (lower panels). (PDF) [file pone.0032276.s002.pdf]

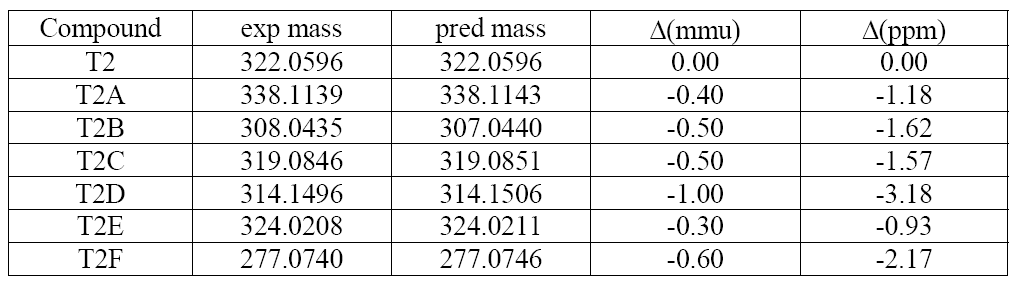

Supplement: Table S10 — Positive ESI Mass Spectra analysis. Accurate mass of [M+H]+ ions. (TIF) [file pone.0032276.s012.tif]
