# Supplementary material for: Diphenyl Urea Derivatives as Inhibitors of Transketolase: A Structure-Based Virtual Screening
Source: PLoS One. 2012 Mar 5;7(3):e32276. doi: 10.1371/journal.pone.0032276 (PMC3293897; doi:10.1371/journal.pone.0032276)

| | | | | | | | |
|---|---|---|---|---|---|---|---|
| **Sample Name** | EM324 | **Position** | P1-B2 | **Instrument Name** | Instrument 1 | **User Name** | |
| **Inj Vol** | 2 | **InjPosition** | | **SampleType** | Sample | **IRM Calibration Status** | Success |
| **Data Filename** | MSD9112g.d | **ACQ Method** | ESIpos.m | **Comment** | T2=O (Sigma ref S715719-1EA) | **Acquired Time** | 6/30/2011 2:22:24 PM |

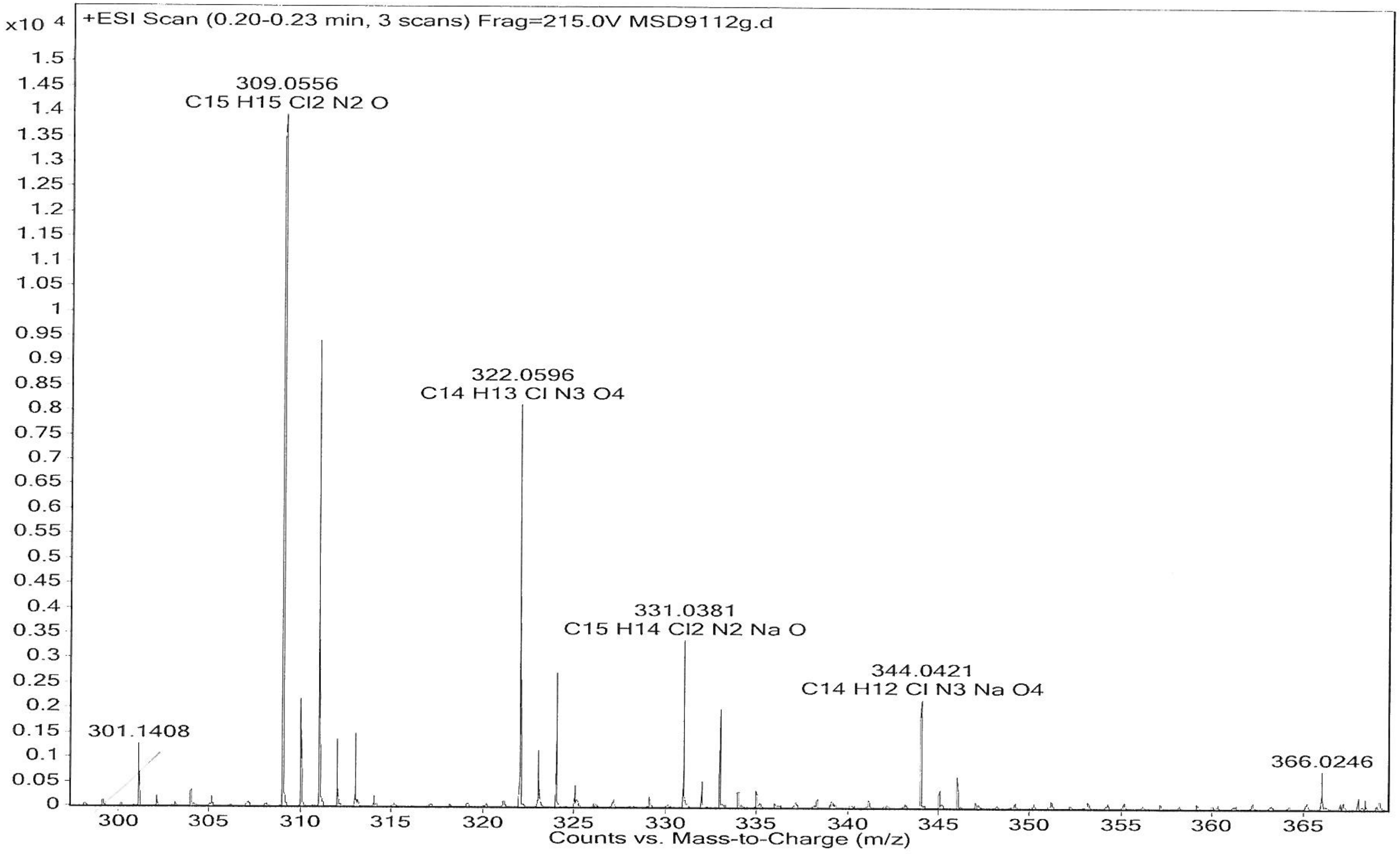

Supplement: Table S3 — Positive ESI Mass Spectra results for T2 compound. (PDF) [file pone.0032276.s005.pdf]
