# Supplementary material for: Diphenyl Urea Derivatives as Inhibitors of Transketolase: A Structure-Based Virtual Screening
Source: PLoS One. 2012 Mar 5;7(3):e32276. doi: 10.1371/journal.pone.0032276 (PMC3293897; doi:10.1371/journal.pone.0032276)

**Sample Name**	EM328	**Position**	P1-B7	**Instrument Name**	Instrument 1	**User Name**	
**Inj Vol**	0.2	**InjPosition**		**SampleType**	Sample	**IRM Calibration Status**	Success
**Data Filename**	MSD9118.d	**ACQ Method**	ESIpos.m	**Comment**	T2E=O3 (Scientific Exchange M-620773)	**Acquired Time**	6/30/2011 5:59:42 PM

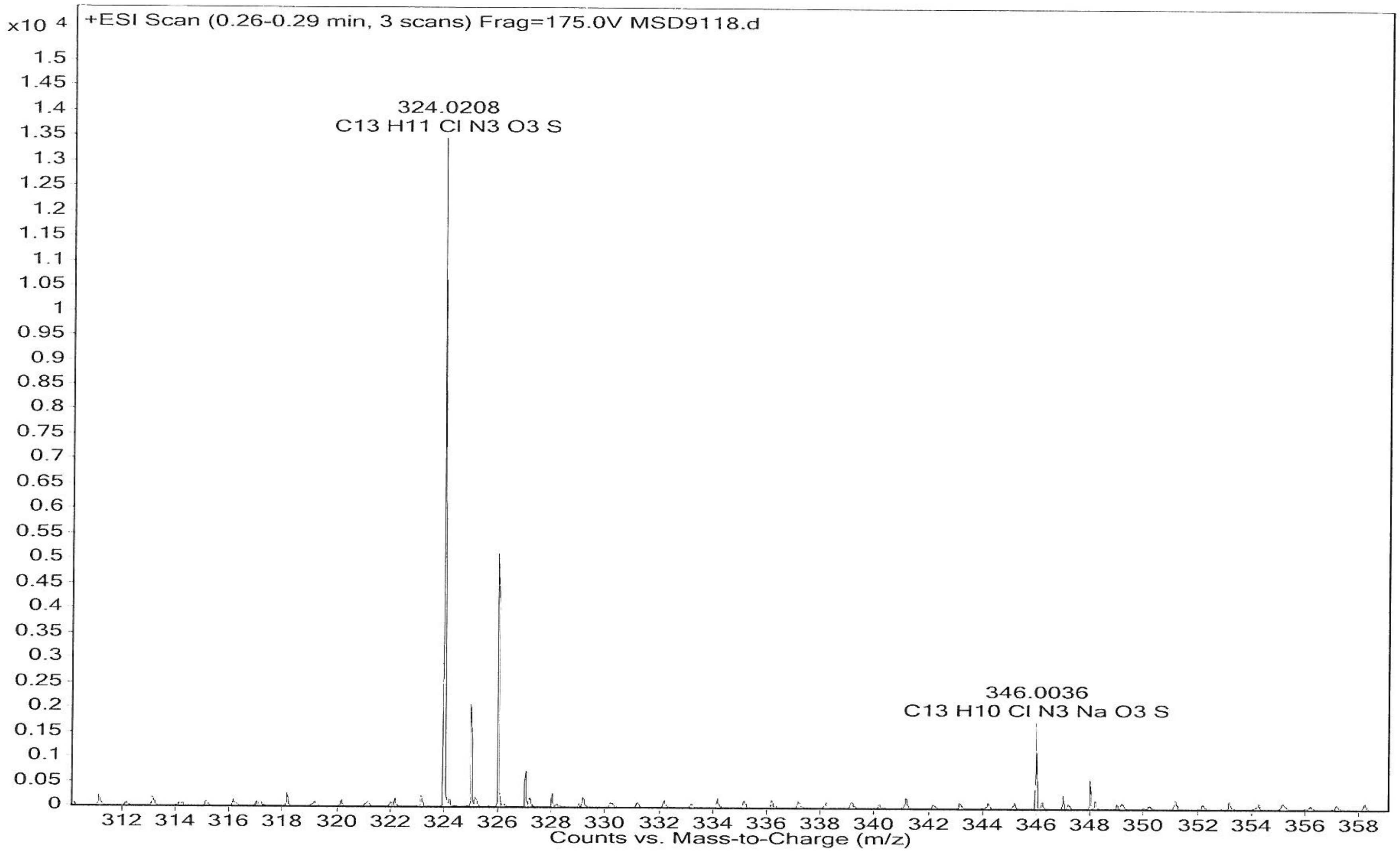

Supplement: Table S8 — Positive ESI Mass Spectra results for T2E compound. (PDF) [file pone.0032276.s010.pdf]
